# Editorial: Advanced methods, equipment and platforms in precision field crops protection, volume II

**DOI:** 10.3389/fpls.2025.1704271

**Published:** 2025-10-07

**Authors:** Zhaoyuan Zhang, Ning Yang, Peter Yuen, Jingcheng Zhang, Jun Ni

**Affiliations:** ^1^ School of Electrical and Information Engineering, Jiangsu University, Zhenjiang, Jiangsu, China; ^2^ Centre for Defence Engineering and Physical Science, Cranfield University, Bedfordshire, United Kingdom; ^3^ School of Automation, Hangzhou Dianzi University, Hangzhou, Zhejiang, China; ^4^ Laboratory of Intelligent Control and Robotics, Shanghai University of Engineering Science, Shanghai, China

**Keywords:** precision perception, crop protection, intelligent decision-making and control, multi-source data fusion, smart agriculture, UAVs

## 1 Introduction

The proliferation of pests, diseases, and weeds constitutes a primary constraint on agricultural productivity. Recently, leveraging modern information technology to realize precision crop protection has become a key area of research within the domain of smart agriculture. This Research Topic focuses on novel sensor technologies for early detection and identification of biotic stresses, artificial intelligence-based methods for intelligent diagnostics and phenotypic analysis, and the development of precision equipment and systems for variable-rate crop protection strategies. Concurrently, this Research Topic delves into the innovative integration of digital twin models, the Internet of Things (IoT), and cloud-based platforms into crop protection paradigms, while also offering a perspective on the future trajectories of this research field.

## 2 Research topic overview

We have compiled a collection of two review articles and fifteen original research studies focusing on the focal points of this Research Topic. The authors’ contributions predominantly encompass the following research domains: the intelligent perception and identification of plant pests and diseases; automation in agricultural operations and precision control systems; the precise quantification of plant phenotypes and disease severity using deep learning; autonomous sensing for intelligent farm machinery coupled with lightweight model optimization; the application of Unmanned Aerial Vehicles (UAVs) for crop disease detection; and the development of a macro-scale agricultural decision support system leveraging multi-modal data fusion. This editorial elucidates the significant progress in intelligent monitoring and precision protection for field crops, underscoring the transformative influence of these modern technologies on global agriculture.

## 3 Precision sensing and monitoring of crop diseases

In crop protection, precision perception and monitoring are fundamental steps, encompassing tasks such as pest and disease identification, disease severity assessment, and crop phenotype extraction. Qiao et al. proposed a method for 3D crop reconstruction and parameter extraction that combines Neural Radiance Fields (NeRF) with a lightweight point cloud segmentation network. This study achieved high-precision segmentation in maize 3D reconstruction, and the proposed method outperformed five existing mainstream networks. Concurrently, the maize stem thickness, plant height, and leaf parameters extracted by this method showed high consistency with manual measurements, demonstrating its reliability and applicability. Turning to wheat stripe rust, Qin et al. introduced a severity assessment method based on lesion expansion. Through experiments with nine method combinations, the authors found that the optimal combination achieved an accuracy of 96.16% in severity assessment—significantly outperforming traditional visual methods and non-expansion approaches—and resolved the discrepancy between grading standards and actual lesion areas. A review by Zhu et al. revealed that Unmanned Aerial Vehicles (UAVs) equipped with multispectral, RGB, and thermal imaging sensors, when integrated with deep learning algorithms, can achieve rapid pest and disease identification at the field scale with accuracy significantly higher than traditional manual surveys. For instance, deep convolutional networks exceeded 95% accuracy in detecting maize leaf blight, whereas manual visual inspection was only approximately 80% accurate. Furthermore, Zhu et al. noted that Large Vision and Language Models (LVMs/LLMs) exhibit “zero-shot” and “few-shot” learning capabilities in agricultural multi-modal data fusion, holding promise for substantially enhancing monitoring performance in agricultural scenarios that lack large-scale annotated data. Their systematic review emphasized the potential of these models in understanding remote sensing images and generating agricultural decisions. The perception and monitoring of pests, diseases, and weeds is evolving from 2D imaging to 3D modeling, lesion quantification, and large model-driven multi-source fusion, thereby greatly enhancing the accuracy and automation of crop health monitoring.

## 4 Intelligent decision-making and control for crop protection

Building upon monitoring data, intelligent decision-making and control technologies facilitate proactive and intelligent crop protection by predicting and identifying potential threats. He et al. proposed the deep learning-based time-series forecasting models, SADF-Net and the RAADA network, which fuse satellite imagery, sensor data, and meteorological data to enable early warnings for crop disease risks. This approach achieved significantly higher temporal forecasting accuracy than LSTM and GRU models, with an improvement of approximately 8%–10% in both prediction accuracy and stability. Wei et al. introduced the IMSFNet+AROS framework, which performs multi-modal anomaly detection using data from UAVs, satellites, and ground-based sensors to aid in the early discovery of in-field anomalies. In the domain of intelligent control for agricultural equipment, Chen et al. developed the YOLOv8-PSS, a lightweight obstacle detection model. Compared to the original YOLOv8, this model reduced the number of parameters by 55.8% and computational overhead by 51.2%, while maintaining a mean Average Precision (mAP) of 90.6%. Localization error was controlled within a range of 2.73%–4.44%, significantly enhancing the safety of unmanned agricultural machinery in complex field environments. Meanwhile, research by Zhang et al. demonstrated that by improving the IPSO-SVM algorithm and fuzzy logic, it is possible to achieve fault prediction and adaptive speed regulation for unmanned combine harvesters, thereby enhancing the equipment’s operational stability and level of intelligence. In summary, intelligent decision-making and control are progressively actualizing a closed-loop management paradigm of “prediction-diagnosis-control,” providing a reliable foundation for proactive defense and intelligent execution in crop protection.

## 5 Precision operations and intelligent equipment

Operation optimization is critical for translating monitoring and decision-making outcomes into practical field applications. Current research primarily focuses on the optimization of Unmanned Aerial Vehicle (UAV) spraying and the mechanisms of droplet deposition. In the field of UAV spraying, the ACHAGA algorithm proposed by Zhang et al. was found to optimize UAV flight paths in complex tea plantation terrains, reducing flight distance and the number of turns. This approach improved efficiency by approximately 20%–30% compared to manual planning, thereby enhancing crop protection efficacy. A study by Yu et al. demonstrated that different flight altitudes and droplet sizes significantly impacted spray deposition distribution on banana canopies, with an altitude of 4 m and a droplet size of 100 μm achieving optimal deposition. This proves the significant influence of operational parameters on deposition uniformity and penetration. Liu et al. optimized UAV spraying parameters for the control of the fall armyworm (Spodoptera frugiperda), showing that a control efficacy of over 90% was achieved using an XR110015VS nozzle, a spray volume of 37.5 L/ha, and a flight height of 2.5 m. This performance was comparable to that of traditional knapsack sprayers but with significantly reduced pesticide dosage and labor intensity. Wind tunnel experiments by Gao et al. indicated that the critical wind speed for droplets on curved leaf surfaces under airflow is significantly correlated with droplet diameter and leaf curvature. As leaf curvature increases, droplets are more easily dislodged, with the difference reaching 24.8%. Furthermore, the acceleration difference for large droplets can be as high as 68%, revealing the influence of airflow and leaf structure on droplet deposition. Meanwhile, research by Wang et al. on targeted spraying in wheat fields showed that the detection accuracy of their improved YOLO model reached 95.6% during the tillering stage—an improvement of 7.3% over the original YOLOv5. This also led to a 40% reduction in pesticide usage, indicating that intelligent spraying can effectively lower the environmental burden while ensuring control efficacy. In conclusion, research on equipment optimization not only enhances the adaptability of UAVs in complex field environments but also promotes improvements in pesticide use efficiency, achieving the goal of “high efficacy with low pesticide volume” in crop protection.

## 6 Conclusion

In summary, current research has established an integrated technological chain for crop protection, progressing from precision perception and monitoring to intelligent decision-making and control, and culminating in precision operation and equipment optimization. At the perception level, a significant leap has been made from 2D imaging to 3D reconstruction, with large models enhancing the capacity for multi-source data fusion. At the decision-making level, artificial intelligence algorithms have substantially improved the accuracy of prediction and diagnosis, enabling autonomous operation of agricultural machinery in complex environments. Subsequently, at the operational level, advancements in UAV path planning and spray parameter optimization have markedly increased pesticide use efficiency and operational throughput. Nevertheless, several challenges persist, including insufficient real-time processing capabilities, limited model generalizability, and the need for enhanced equipment stability in complex operational settings. Future research should therefore focus on the deeper integration of advanced sensors, intelligent algorithms, and digital twin platforms to construct a more efficient, environmentally friendly, and intelligent system for field crop protection.

